# Melatonin receptors and Per1 expression in the inferior olivary nucleus of the *Sapajus apella* monkey

**DOI:** 10.3389/fnins.2022.1072772

**Published:** 2022-12-20

**Authors:** Marcos Donizete Junior Granado, Luciana Pinato, Jeferson Santiago, Sandra Maria Barbalho, Jessica Ellen Lima Parmezzan, Lenita Mayumi Suzuki, Mayara Longui Cabrini, Daniele Raineri Mesquita Serva Spressão, Ana Letícia Carneiro de Camargo, Leila Maria Guissoni Campos

**Affiliations:** ^1^Postgraduate Program in Structural and Functional Interactions in Rehabilitation, Medical School, University of Marilia (UNIMAR), Marília, Brazil; ^2^Department of Speech, Language and Hearing Sciences, São Paulo State University (UNESP), Marília, Brazil

**Keywords:** biological rhythms, melatonin, clock genes, primate, motor

## Abstract

Melatonin is a transducer of photic environmental information and participates in the synchronization of various physiological and behavioral phenomena. Melatonin can act directly in several areas of the central nervous system through its membrane receptors coupled to G protein, called MT1 and MT2 receptors. In some structures, such as the retina, hypothalamus and pars tuberalis, the expression of both melatonin receptors shows circadian variations. Melatonin can act in the synchronization of the clock proteins rhythm in these areas. Using the immunohistochemistry technique, we detected the immunoexpression of the melatonin receptors and clock genes clock protein Per1 in the inferior olivary nucleus (ION) of the *Sapajus apella* monkey at specific times of the light-dark phase. The mapping performed by immunohistochemistry showed expressive immunoreactivity (IR) Per1 with predominance during daytime. Both melatonin receptors were expressed in the ION without a day/night difference. The presence of both melatonin receptors and the Per1 protein in the inferior olivary nucleus can indicate a functional role not only in physiological, as in sleep, anxiety, and circadian rhythm, but also a chronobiotic role in motor control mechanisms.

## Graphical Abstract

Inferior olivary nucleus: Melatonin receptors and Per1. The presence of both melatonin receptors, as well as the Per1 clock protein in the inferior olivary nucleus, is an indicator for a chronobiotic role in motor control mechanisms. 
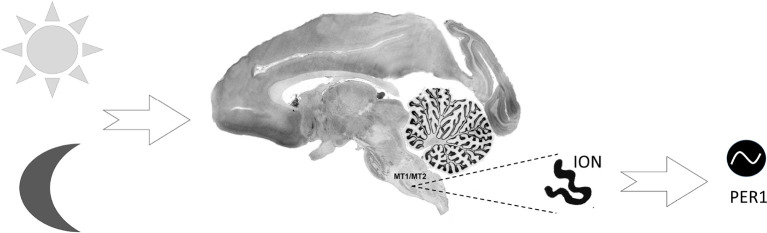

## 1 Introduction

Melatonin, produced rhythmically by the pineal gland, presents high circulating levels during the night and regulates several physiological and neuroendocrine functions through two G protein-coupled receptors (GPCRs), the MT1 and MT2 receptors, which are widely expressed in different tissues (Lacoste et al., 2015; Ng et al., 2017; Pinato et al., 2017). Both receptors are thus involved in the physiology and pathologies of different systems, including the central nervous system (CNS) (Dubocovich et al., 2010; Tosini et al., 2014; Pinato et al., 2015; Jockers et al., 2016; Hardeland, 2018).

In CNS, the MT1 and MT2 receptors have already been described with substantial regional differences in expression levels in the hypothalamus, thalamus (Thomas et al., 2002; Gupta et al., 2013; Odo et al., 2014; Pinato et al., 2017), cerebral cortex, putamen, caudate nucleus, nucleus accumbens, amygdala, hippocampus, septal area, striatum, ventral tegmental area, habenula (Uz et al., 2005; Lacoste et al., 2015), and pars tuberalis (Schuster et al., 2001; Wu et al., 2006; Lacoste et al., 2015).

The expression of melatonin receptors can be rhythmic in some of these areas and present some variability in different species (Gupta et al., 2013; Odo et al., 2014; Pinato et al., 2017). This vast expression in the CNS indicates the involvement of both receptors in the mechanisms underlying different brain functions, whether under the action of endogenous or exogenous melatonin and melatonergic agonists (Zlotos et al., 2014; Liu et al., 2016).

The mammalian circadian clock system is based on cellular oscillators in all body tissues organized hierarchically. A master pacemaker located in the suprachiasmatic nucleus (SCN) synchronizes peripheral tissue clocks and extra-SCN oscillators in the brain with each other and with external time. Different time cues (so-called Zeitgebers) such as light, food intake, activity, and hormonal signals reset the circadian clock system through the SCN or by direct action at the tissue clock level. Several extra-SCN central oscillators were characterized regarding circadian rhythm regulation and output. Some are directly innervated by the SCN pacemaker, while others receive indirect input from the SCN via other neural circuits or extra-brain structures (Begemann et al., 2020).

With a degree of essentiality varying among species, the role of melatonin in the control of circadian phenomena is in regulating the circadian phase and maintaining rhythm stability of SCN neurons (Reppert et al., 1988; Bell-Pedersen et al., 2005; Pevet and Challet, 2011; Pfeffer et al., 2012; Adamah-Biassi et al., 2014). In this process is included the induction of Per1 mRNA expression by melatonin (Weaver et al., 1989; Gillette and McArthur, 1996; Dubocovich et al., 2003) influencing at least the sleep/wake cycle and the circadian rhythm of locomotor activity (Dubocovich et al., 2005).

Several extra-SCN central oscillators were characterized regarding circadian rhythm regulation and output. Some of them are directly innervated by the SCN pacemaker, while others receive indirect input from the SCN via other neural circuits or extra-brain structures. The specific physiological function of these non-SCN brain oscillators and their role in regulating the circadian clock network remains understudied (Begemann et al., 2020).

The Per1 and other clock genes are rhythmically expressed in other brain areas (Masubuchi et al., 2000; Campos et al., 2015; Rawashdeh et al., 2018) where melatonin can modulate this rhythmic expression through its receptors such as demonstrated for clock gene Per1 expression in the pituitary gland (von Gall et al., 2002), Per1, Per 2, Bmal1 and Cry 1 in the Pars tuberalis (Jilg et al., 2005), and Per1 expression in the striatum (Uz et al., 2003).

There is evidence for the emerging concept of melatonin receptor dysfunction and the changes in the clock gene expression as a permissive condition favoring the development and/or progression of neurodegenerative diseases. Altered expression of melatonin receptors or clock genes has been frequently reported in neurodegenerative diseases and psychiatric disorders, including Alzheimer’s disease (AD) (Wu et al., 2007; Cronin et al., 2017), Parkinson’s disease (PD) (Adi et al., 2010; Li et al., 2017), and Huntington’s disease (HD) (Pallier et al., 2007; van Wamelen et al., 2013).

On the other hand, the neuroprotective effect of endogenous and exogenous melatonin has been demonstrated in several neurodegenerative conditions, including amyotrophic lateral sclerosis (Weishaupt et al., 2006; Zhang et al., 2013), PD (Naskar et al., 2015), and HD (Escribano et al., 2014).

The requirement of melatonin receptors for the neuroprotective action of melatonin has also been demonstrated in a series of studies (Lee et al., 2010; Wang et al., 2011; Pinato et al., 2015).

Melatonin would participate in the movement initiation process due to the probable inhibition of dopamine secretion through the MT1 receptor (Zisapel, 2001). Besides this, little was described as melatonin receptors and their relationship with clock genes in brain areas related to motor functions. Both receptors and clock genes were found in the cerebellum (Guissoni Campos et al., 2018), where the maximal expression of melatonin receptors was found to coincide with that of Per proteins, base nuclei, and the substantia nigra (Uz et al., 2003, 2005; Christiansen et al., 2016).

Considering the expression and localization of melatonin receptors as the basis for investigations of their role in neuronal functions, and that melatonin was one of the most effective candidates for preventing neuronal death in several pathologies, we investigated the day/night expression of the Per1 protein, MT1, and MT2 melatonin receptors in the inferior olivary nucleus (ION), essential in processing motor learning, control and coordination of movements (Rasmussen and Hesslow, 2014; Najac and Raman, 2015) thought connections to the cerebellum (Reeber et al., 2013; Rasmussen and Hesslow, 2014; Najac and Raman, 2015).

The results expand the repertoire of neuroanatomical knowledge of these two pharmacological targets. The availability of this information can contribute to further advances in the therapeutic of motor diseases.

## 2 Materials and methods

In the present study, slices of the brains of six adult male tufted capuchin monkeys (*Sapajus apella*) (2 to 3 kg) of the same age, and weight, without visible motor alterations, without a history of previous diseases, in physiological condition were obtained from the Center of Tufted Capuchin Monkey Procreation of the São Paulo State University (UNESP), Araçatuba, SP, Brazil. The animals were kept in individual cages under natural conditions of light (with dusk and dawn natural light conditions), temperature and humidity during the experiments and fed with a controlled diet consisting of eggs, fruit, granulated ration with protein, and dried corn; water was provided *ad libitum*. In this specific season, the sunrise time during the experiments was approximately 06:00am and was considered the Zeitgeber time 0 (ZT0) as a reference; the sunset time started at approximately 06:00pm.

Following this time parameter, the animals were anesthetized and perfused at ZT 0, (day point) and ZT 15 (night point), *N* = 3 per ZT. The procedures involving animal use were compliant with the “Guidelines for the care and use of mammals in neuroscience and behavioral research” (2003) and were approved by the local ethics committee (process n*^o^* 2013-00259/FOA-UNESP).

### 2.1 Animals

Analyzes of Per1, MT1, and MT2 immunoreactive cells were performed in six capuchin monkeys (*Sapajus apella*) (*N* = three per ZT). The animals were perfused following the protocol described by Campos et al. (2014) with 0.9% saline solution and 4% paraformaldehyde. After perfusion, the brains were exposed and cut into blocks using a stereotaxic apparatus. The blocks were removed from the skull and placed in a cryoprotective solution containing 10% glycerol and 2% dimethylsulfoxide in a 0.1 M borate buffer, pH 9.0, at 4°C. After three days, the blocks were transferred to a similar solution containing an increased concentration of glycerol (20%). They were incubated for four additional days, as previously described (Rosene et al., 1986). After cryoprotection, the brain blocks were cryosectioned into 30 μm-thick sections using a cryostat (Leica CM 1850, Microsystems AG, Germany) and stored as 10 different stepwise series in anti-freeze solution until the time of immunohistochemical processing or Nissl staining.

### 2.2 Immunohistochemistry

Brain sections were processed using immunohistochemical techniques for melatonin receptors and Per 1. The sections were washed using a solution TBS-TX buffer (0.05 M), incubated for 48 h at 4°C in a solution containing 0.05 M TBS-TX buffer, 2% normal serum (Vector Laboratories, USA), and the appropriate primary antibody: anti-MT1 (1: 200, Santa Cruz), anti-MT2 (1: 200, Santa Cruz), anti-Per1 (1:500, Santa Cruz, USA) separately. Next, the sections were washed with 0.05 M TBS-TX and incubated in a secondary antibody Alexa 488 (1:200, *Jackson* Immuno Research) and Cy3 (1:200, *Jackson* Immuno Research) fluorescents specific for the primary antibody species, diluted (1:200) in the same solution that the primary, for two hours.

### 2.3 Data analysis

The ION was identified using brain sections stained with Nissl and the atlases “A Stereotaxic Atlas of the Brain of Cebus Monkey” (*Cebus apella*) (Manocha et al., 1968) and “The Rhesus Monkey Brain in Stereotaxic Coordinates” (Paxinos et al., 2000). The DAPI (Sigma Chemical) fluorescent staining methodology and Nissl staining were used to identify the brain area. For each animal, all the coronal sections of a series representing the whole extension of the ION were placed in a rostrocaudal order. After that, three sections of each animal, similar across animals (representing the same rostrocaudal level), were processed for each antibody. The sections representing different levels of the rostrocaudal extension were adjacent among antibodies. Each coronal section was analyzed under a light field (Olympus BX50 microscope), and the images were acquired with cellSens software (USA). The image was obtained with adequate resolution, and evenly brightness and contrast were changed using Adobe Photoshop CS6. Schematic drawings were performed using the Canvas 6 software (Deneba, USA). Cell Counter plugin – ImageJ (National Institutes of Health, USA) was applied to count the number of neurons of the two ZTs. The manual cell counting tool was used from a single color fluorescence image-RGB color by clicking on the cell image. Each click marks the cell with a colored square and adds the cell to a tally sheet. Labeling intensity was measured by the optic density (OD) analysis of the – ImageJ.

### 2.4 Statistical analyses

The data are expressed as the mean ± standard error of the cell number of the three monkeys perfused in the same ZT. The data are also presented as the mean ± standard error of the relative number of neurons by the surface area of the nucleus slices. The Mann-Whitney test or the Student’s t-test was applied to compare the two ZTs. Values of p < 0.05 were considered statistically significant.

## 3 Results

**FIGURE 1:**
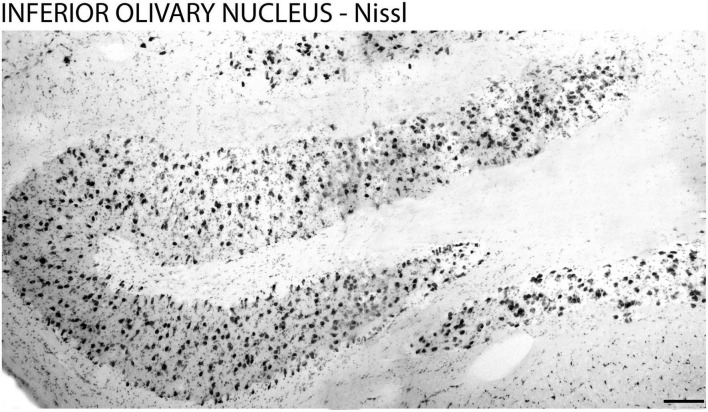
Cytoarchitecture of the inferior olivary nucleus in the brain of the primate *Sapajus apella*. Photomicrograph of the frontal section of the brain of the primate *Sapajus apella* stained with Nissl. Bar = 100 μm.

**FIGURE 2:**
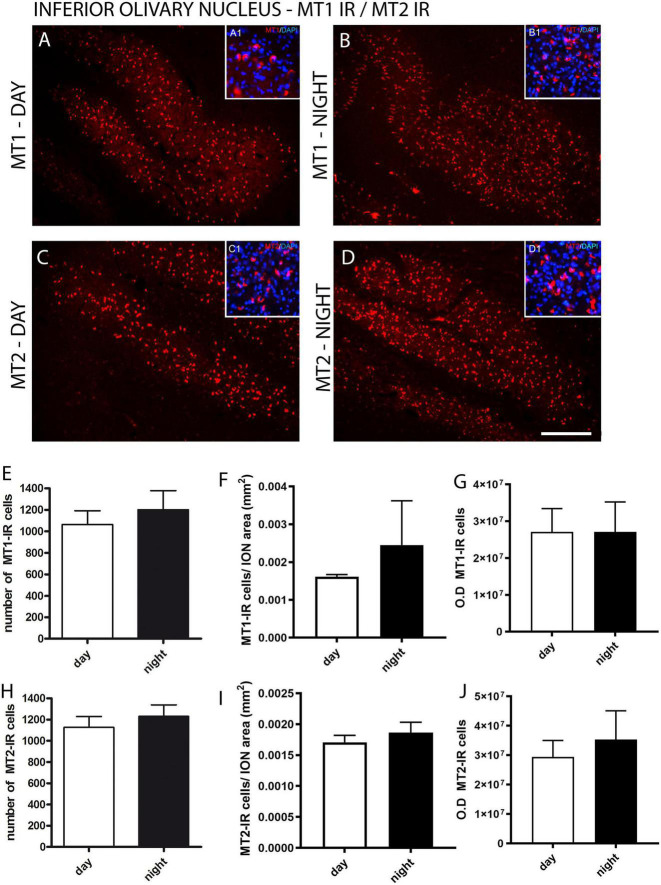
Distribution of MT1 and MT2 receptors in the inferior olivary nucleus (ION) of the primate *Sapajus apella*. Immunofluorescence photomicrographs of frontal sections of the primate’s brain *Sapajus apella* showing MT1-labeled cells (MT1-IR) in the ION day and night **(A,B)** and MT2-IR cells day and night **(C,D)**. In **(E,H)**, the number of MT1 or MT2 immunoreactive cells in the ION. The relative number of day and night MT1-IR and MT2 cells by the surface area of the ION **(F,I)**. The intensity of staining day and night MT1-IR and MT2 cells **(G,J)** - optical density (OD). Photomicrographs of the cytoarchitecture characterization of neuronal populations of the inferior olivary nucleus (ION) represented by fluorescent staining in DAPI (blue) in A1, B1, C1, D1. Bar = 100 μm.

The cytoarchitecture and the boundaries of the ION were evidenced by Nissl staining (Figure 1) and fluorescent DNA marker (DAPI) (Figures 2A1-D1) which allowed the identification of the design of the ION.

For the fluorescent immunohistochemistry of melatonin receptors, the analyses showed similar results in the immunoexpression between the MT1 and MT2 receptors in the ION between day and night (Figures 2A–J). There was no difference (p = 0.53) between the number of MT1-IR cells quantified during the day (2413 ± 348.3) and night (2840 ± 144.3) and between the number of MT2-IR cells (*p* = 0.59) quantified during the day (2793 ± 277.5) and night (2533 ± 389.6) (Figures 2E, H). No difference was found either between day and night in the relative number of MT1-IR or MT2-IR cells by the surface area of the ION (MT1 day 0.0016 vs. MT1 night 0.0024, p = 0.20; MT2 day 0.0017 vs. MT2 night 0.0018, p = 0.20) (Figures 2F, I). Also, when the staining intensity was investigated, there was no difference between day and night MT1-IR and MT2-IR (Figures 2G, J).

**FIGURE 3:**
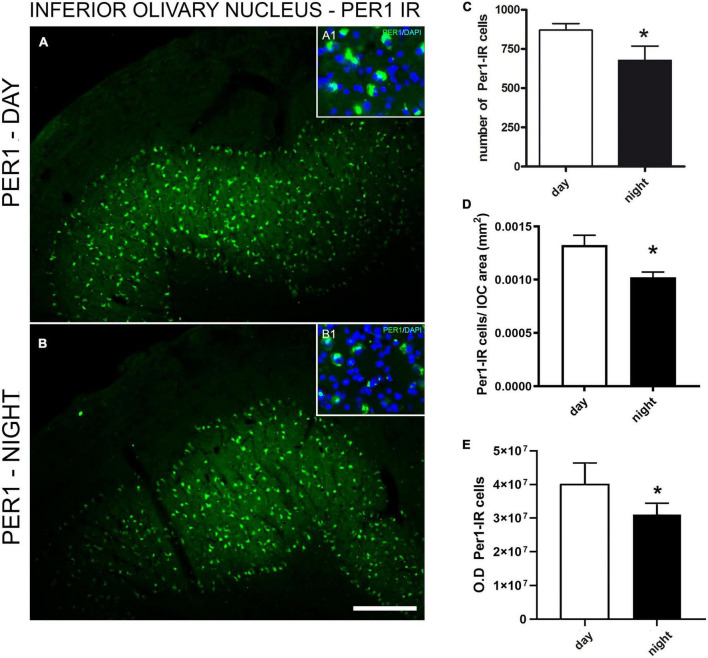
Immunofluorescence photomicrographs of the frontal section of the inferior olivary nucleus (ION) of the primate *Sapajus apella* showing Per1 – IR cells in **(A)**. Mean of the number of immunoreactive cells to Per1 in 3 brain sections representative of anteroposterior extension of the inferior olivary nucleus (ION) of the primate *Sapajus apella* (*N* = 3 ZT). Photomicrograph of the cytoarchitecture characterization of neuronal populations of the ION represented by fluorescent staining in DAPI (blue) in A1 and B1, overlapping with A. Per-1 antibody showed the specificity of labeling in neuronal populations, allowing the delineation of the ION **(A,B)**. Per1 in neuronal populations of the ION **(C)**. Relative number of day and night Per1-IR cells by the surface area of the ION **(D)**. Intensity of staining - OD **(E)**. Bar = 100 μm.

The Per1 immunoexpression was observed in the anteroposterior levels of the ION, but not in adjacent areas, demonstrating the specificity of Per1 in neuronal populations of the ION (Figure 3). A larger amount of Per1–IR cells was observed in the daytime (870.8 ± 40.8) than in the night (677.3 ± 91.7) in the ION (Figure 3C). The same day-night difference was found between the relative number of Per1-IR cells by the surface area of the ION (day 0.0013 vs. night 0.0010, p = 0.05) (Figure 3D). The difference (p = 0.01) between day (40083863 ± 2539390) and night (31056641 ± 1330252) Per1 expression was also found when the intensity of staining was investigated (Figure 3E).

## 4 Discussion

The present study demonstrated MT1 and MT2 melatonin receptors and Per1 immunoreactivity in ION in a physiological condition. It was the first description of the presence of both receptors in the ION of a primate and one of the few who described melatonin receptors in motor areas. Even though the functional and pharmacological role of melatonin receptors is still unclear in motor areas, this finding indicates that in a health condition, both melatonin receptors are present and can be a target for melatonin in ION.

Abnormal expression of the MT1/MT2 receptor has been reported in several neurological diseases (Savaskan et al., 2001; Wang et al., 2011). In some experimental studies of neurodegenerative diseases with motor deficits, like in genetic models of HD (Wang et al., 2011) or amyotrophic lateral sclerosis (Zhang et al., 2013), it was demonstrated that disease progression was associated with the loss of both melatonin and the MT1 receptor in striated and spinal cord respectively. In these situations, exogenous melatonin can attenuate the down-regulated expression of MT1 and neuronal death. Also, MT1 has been shown to protect C6 astroglial cells from oxidative damage and excitotoxicity (Das et al., 2008).

The ION has long been considered major cerebellum afferent from the brain stem. Besides being strongly connected to the brain, integrating both somatic and visceral messages to the cerebellum and playing an important role in the control of movement and the processing of motor learning (Reeber et al., 2013; Rasmussen and Hesslow, 2014; Najac and Raman, 2015).

The results showed no day-night difference between MT1-IR and MT2-IR in the ION. The lack of rhythmicity does not rule out these receptors like targets in treating diseases involving the ION (Stankov et al., 1994; Fraschini et al., 1999). Previously, these receptors had already been described in the cerebellum, another area known to be involved in motor control (Mazzucchelli et al., 1996; Guissoni Campos et al., 2018). The presence of these receptors in this nucleus reinforces the possible melatonin participation in controlling circadian phenomena subserving motor performance (Stankov et al., 1994), in addition to other functions already described (Reiter et al., 2012; Cipolla-Neto et al., 2014).

In the present study, the expression of Per1-IR was higher in the daytime than in the nighttime point analyzed in ION of *Sapajus apella*, similar to the result observed in the cerebellum of the same species (Guissoni Campos et al., 2018). Previous studies have demonstrated in rats and mice a peak of Per1proteins and *Per1 genes* expression at night (Mendoza et al., 2010).

Possibly this difference may be related to the difference in the activity habits of the diurnal primate *Sapajus apella*. Moderate levels of Per1 expression were detected in many brain regions, including the granular layer of the cerebellum, anterior paraventricular thalamic nucleus, caudate-putamen, inferior colliculus, pontine nuclei, ION, and nucleus of the solitary tract (Masubuchi et al., 2000; Campos et al., 2015; Rawashdeh et al., 2018).

Although the mechanism responsible for conferring information on circadian time from the SCN to the ION remains enigmatic, external cues such as feeding schedule, neurotransmitters and neurohormones have been shown to entrain extrahypothalamic oscillators in the brain (Verwey and Amir, 2009). The presence of Per1 in the ION may indicate a circadian action associated with local motor function. Since the expression of clock genes found in the caudate and putamen nuclei of rats has already been correlated to locomotor activity (Masubuchi et al., 2000).

The present study’s data establish characteristics that can impact how information is processed in the ION due to day/night changes in clock protein Per1. The presence of melatonin receptors in the ION showed a possible target for melatonin or its agonists in this area and also indicated that melatonin can be involved in the modulation of clock genes expression in this motor area as demonstrated in other brain areas, as pars tuberalis, and SCN (Coelho et al., 2015; Kandalepas et al., 2016).

The limitation of the study is the number of periods and the number of animals in each period which is common in non-human primate studies. Comparing the results obtained in this typical study with non-human-primate with other models of pathologies will enable advances in scientific knowledge in this area.

## Data availability statement

The data and any supplementary material related to this article can be obtained from the corresponding author on request.

## Ethics statement

The animal study was reviewed and approved by the Brazilian College of Animal Experimentation (COBEA) and the local ethics committee (Protocol Araçatuba Dental School/São Paulo State University – ADE/UNESP process no. 00259/2013).

## Author contributions

MG, LG, and LP contributed to the conception and design of the study. JS, LS, JP, AC, MC, and DS performed the experiments. JS, LS, JP, and LG analyzed the data. MG, LP, SB, and LG drafted the manuscript. LG and LP critically reviewed the manuscript. All authors contributed to the article and approved the submitted version.
